# A Multimodal Eye Assessment in Psoriatic Arthritis Patients *sine*-Psoriasis: Evidence for a Potential Association with Systemic Inflammation †

**DOI:** 10.3390/jcm9030719

**Published:** 2020-03-06

**Authors:** Maria Sole Chimenti, Paola Triggianese, Giorgia Salandri, Paola Conigliaro, Claudia Canofari, Francesco Caso, Luisa Costa, Carlo Nucci, Francesco Aiello, Massimo Cesareo, Roberto Perricone

**Affiliations:** 1 Rheumatology, Allergology and Clinical Immunology, Department of “Medicina dei Sistemi”, University of Rome Tor Vergata, 00133 Rome, Italy; 2 Ophthalmology Unit, Department of Experimental Medicine, University of Rome Tor Vergata, 00133 Rome, Italy; 3 Rheumatology Unit, Department of Clinical Medicine and Surgery, University of Naples Federico II, 80131 Naples, Italy

**Keywords:** dry eye, inflammation, microperimetry, psoriatic arthritis, retinal disease, spectral domain-optical coherence tomography, retinal imaging

## Abstract

Background: Ocular involvement in Psoriatic Arthritis (PsA) patients is mainly associated with uveitis but there remains a paucity of data on dry eye and retinal abnormalities. We aimed to analyze dry eye and subclinical retinal abnormalities in a cohort of PsA patients sine-psoriasis (PsO). Methods: PsA patients sine-PsO were enrolled. Best-corrected-visual-acuity, ocular-surface-disease-index (OSDI), Schirmer test, tear film breakup-time, standard-automated-perimetry (SAP, mean deviation—MD, pattern standard deviation—PSD), fundus-perimetry (FP), and spectral-domain-optical-coherence-tomography (SD-OCT) were performed. Results: A total of 80 eyes from 40 PsA patients with moderate-severe disease activity, and 70 eyes from 35 healthy control (HC) were evaluated. Higher dry eye prevalence occurred in PsA than HC (*p* < 0.0001). ESR was positively related with OSDI (*p* < 0.001) and negatively related with Schirmer (*p* = 0.007). In PsA, SAP registered higher MD (*p* < 0.0001) and higher PSD (*p* = 0.005) in comparison with HC. PSD resulted positively correlated with ESR (*p* = 0.04) and CRP (*p* = 0.01), while MD showed a negative correlation with CRP (*p* = 0.01). Both FP mean differential sensitivity and mean defect were lower in PsA then HC (*p* < 0.0001). In PsA, FP differential sensitivity was directly related with cumulative steroids (*p* = 0.02). Conclusions: In PsA patients sine-PsO, dry eye and subclinical abnormalities in visual functions occurred being potentially related to systemic inflammation.

## 1. Introduction

Psoriatic arthritis (PsA) is a chronic immune-mediated disease belonging to the group of spondyloarthritis (SpA) commonly associated with psoriasis (PsO) [1]. Inflammatory processes involve entheses, joints, digit, tendons, axial skeleton, skin, and nails [2,3]. On the basis of the clinical expression of the two main components of the disease (skin and joint manifestations), we can distinguish PsA with clinically evident skin or nail PsO, and PsA sine-PsO, as patients with family history of PsO [4].

In the last years, several studies have described PsA as a systemic condition and ocular involvement has been reported as a possible related manifestation [5].

Psoriatic arthritis related inflammatory ocular manifestations may affect multiple ocular structures and can occur in up to 32.63% of patients [6]. In particular, anterior uveitis is the most common ocular manifestation associated with PsA (estimated prevalence 2–25%) [7]. Uveitis can lead to be responsible for visual loss, vision impairment, and sometimes the uveitis onset can forerun joint and skin diseases in PsA. Alongside this, ophthalmic manifestations have also been described in patients with PsO [8]. Keratoconjunctivitis sicca and retinal microvascular changes are possible ocular manifestations documented in psoriatic disease [8,9].

The presence of PsO may influence the occurrence of dry eye syndrome in PsA patients. As widely documented from both case-series studies and population-based studies, the presence of PsO is related to dry eye disease and its possible complications (as keratopathy) [10,11]. Dry-eye disease, in systemic inflammatory diseases, is associated with elevated levels of pro-inflammatory cytokines in tear film [12,13]

Data concerning dry eye syndrome in PsA patients without PsO is lacking to date.

As previously described, disturbances in the balance of pro-angiogenesis and anti-angiogenesis factors can lead to the progression of vascular damage in several chronic inflammatory conditions with the occurrence of retinal involvement at multiple levels, vascular endothelium changes are strictly involved in PsA pathogenesis [14,15] developing retinal damage [16]. The presence of subclinical retinal involvement can be studied using modern techniques, such as the spectral-domain optical coherence tomography (SD-OCT) and fundus perimetry (FP), also called microperimetry [17].

Spectral-domain OCT (SD-OCT) is a non-invasive and objective imaging test that uses infra-red light waves to take cross-sectional scans of the retina. Fundus perimetry represents a technique for measuring visual field sensitivity whilst simultaneously viewing the ocular fundus [18,19]. The early detection of subclinical abnormalities in retinal morphology and function in PsA patients sine-PsO has not been reported to date.

Aims of this prospective study were to evaluate the ocular discomfort/dry eye syndrome and to explore the prevalence of subclinical retinal involvement in a cohort of PsA patients sine-PsO, in order to avoid the potential influence of cutaneous disease on ophthalmological findings. Furthermore, potential correlations between morphological and functional visual abnormalities and PsA disease activity have been investigated.

## 2. Materials and Methods

### 2.1. Study Population

This non-interventional cross-sectional study evaluated patients with PsA referred to the Rheumatology Outpatients Clinic of the University of Rome Tor Vergata from February 2019 to July 2019. Healthy controls (HC), matching for age and sex, were also recruited at the Ophthalmology Clinic of the University of Rome Tor Vergata in the same timeframe.

Inclusion criteria were: (1) Diagnosis of PsA according to the classification criteria for psoriatic arthritis (CASPAR) [20] with no evidence of skin PsO; (2) patients naïve to systemic treatment; (3) aged ≥18 years old and ≤ 65 years old; (4) intraocular pressure <21 mmHg on diurnal testing measured using Goldmann applanation tonometry; (5) spherical equivalent refractive error between −6.0 and +4.0 diopters; (6) open irido-corneal angle on gonioscopy [21].

Exclusion criteria were: (1) Established primary ocular diseases including a history of any retinal or optic nerve disease; (2) presence of any drusen-like deposits, retinal focal atrophy, retinal pigment epithelium detachment and any alterations associated to drug toxicity at indirect ophthalmoscopy or SD-OCT examination; (3) lens opacities; (4) systemic comorbidities with ocular involvement; (5) other autoimmune systemic conditions; (6) systemic treatments that may affects quantity or quality of tear film. Patients were also excluded if they presented with a glaucomatous optic nerve configuration [21].

Written informed consent was obtained from patients and controls according to the Declaration of Helsinki (updated 2008) and the study was approved by the scientific ethic committee of the University of Rome Tor Vergata, Rome, Italy.

### 2.2. Rheumatologic Assessment

The disease activity in patients with PsA was assessed by an experienced rheumatologist at the time of recruitment using tender (68 joints) and swollen joint (66 joints) count, enthesitis (Yes/No), dactylitis (Yes/No), relevant patient reported outcomes (PROs) such as Visual Analogue Scale of pain (VASp) and global health (VASgh), Bath Ankylosing Spondylitis Disease Activity Index (BASDAI), Health Assessment Questionnaire modified for SpA (HAQ-SpA) [22]. The Disease Activity in Psoriatic Arthritis (DAPSA) score [23] and the Disease Activity Score (DAS) were used as composites clinimetric indexes [24].

We recorded patients’ information including age, body weight, disease duration, medication history regarding corticosteroids use (daily and cumulative doses) and on-demand non-steroidal anti-inflammatory drugs use. All patients had laboratory assays including erythrocyte sedimentation rate (ESR) and C-reactive protein (CRP) performed at the laboratory of the University of Rome Tor Vergata as part of the routine care.

### 2.3. Ophthalmological Assessment

All subjects underwent a standard ophthalmology examination, including best corrected visual acuity (BCVA), Ocular Surface Disease Index (OSDI) questionnaire, Schirmer test, and tear film Breakup Time (BUT). BCVA was measured using a standard LogMAR eye chart according to the Early Treatment of Diabetic Retinopathy Study (ETDRS) protocol. The OSDI Questionnaire was administered to all the subjects included in the study in order to assess dry eye disease symptoms and severity in a scale of normal, mild to moderate, and severe. The ocular surface disease index questionnaire is a validated PRO instrument that provides a measure of the ocular symptoms associated with dry eye. It includes 12 questions relating to past week experience with the presence of ocular discomfort symptoms, vision-related functioning, and environmental triggers [25]. An OSDI score ≤12 was considered normal while a score between 13 and 32 indicated the presence of mild to moderate dry eye and a score ≥33 indicated a severe dry eye [25].

The Schirmer I test was performed in all subjects: A filter paper strip (Alfa Intes, Industria Terapeutica Splendore, Casoria, Italy) was applied between the outer and the middle third of the lower lid. The length of the wetted part of the strip was measured after 5 min: Values lower than 5 mm imply a diagnosis of dry eye [26].

The BUT was performed using a wetted fluorescein strip (Fluorescein sodium ophthalmic strips U.S.P. Alfa Intes Industria Terapeutica Splendore, Casoria, Italy) and the ocular surface was observed at the slit lamp through a cobalt blue filter. The time gap between a blink and the appearance of a dark spot on the cornea was recorded and the mean of three consecutive measurements was reported. Values lower than 10 sec were used for a diagnosis of dry eye [27].

The presence of dry eye was established with the presence of either a quantitative or qualitative disturbance of the tear film (Schirmer test ≤5 mm and/or BUT <10 sec).

### 2.4. Perimetry, Fundus Perimetry, and Spectral Domain Optical Coherence Tomography

Standard automated perimetry (SAP), FP, and SD-OCT scan were performed in all subjects. Standard automated perimetry was performed with a Humphrey Field Analyzer (HFA; model 750, Zeiss Humphrey Systems, Dublin, CA, USA), using the SITA-Standard program 30-2. Mean deviation (MD) and pattern standard deviation (PSD) were measured by SAP and compared between groups. Abnormalities of visual fields were based on the pattern deviation plots [28].

Fundus perimetry is a subjective functional test that images the retina during visual field testing, enabling a correlation to be made between visual function (i.e., differential light sensitivity threshold values) and retinal structure.

All enrolled subjects underwent FP using 10-2 pattern with a Nidek MP-1 microperimeter (Nidek Technologies Srl., Vigonza, PD, Italy). Fundus perimetry was performed under mydriatric state in a dedicated quiet dark room using Goldmann size III white stimuli presented for 200 ms with a 4-2 staircase threshold strategy. The fixation target used for all subjects was a one grade in diameter red cross on a white, monochromatic background at 1.27cd/m^2^ (4 apostilbs). Mean sensitivity and mean defect values were obtained by the MP1 in-built software (1.7.8—2014-12-10) [29,30].

The posterior pole scanning protocol was performed with SD-OCT Spectralis (Heidelberg Engineering, Heidelberg, Germany) in all patients and HC group, after pupil dilation with mydriatic eye drops (phenylephrine 10% + tropicamide 0.5%, Visumidriatic Fenilefrina, Visufarma, Italy). Images were acquired using the image alignment eye-tracking software (TruTrack; Heidelberg Engineering GmbH) to obtain volumetric retinal scans comprised of 61 single axial scans (scanning area: 30 ° × 25 °) centered on the fovea, with a fovea-to-disc inclination of 7 degrees. No manual correction to the Spectralis automatic segmentation of the different retinal layers was necessary [30].

### 2.5. Statistical Analysis

To test normality of data sets, the D’Agostino and Pearson omnibus test was used. Normally distributed variables were summarized using mean and standard deviation (SD). Non-normally distributed variables were summarized using median with percentile ranges. Continuous variables were compared using the parametric unpaired t-test or the nonparametric Mann–Whitney U test when appropriate. Categorical variables were presented with absolute frequencies and percentages, and were compared using the Chi-squared test or Fisher’ exact test when appropriate. The significance of any correlation was determined by Pearson correlation test or Spearman’s rank correlation coefficient where appropriate. *P* values <0.05 were considered significant. All statistical analyses were performed using GraphPad Prism version 7 (GraphPad software).

## 3. Results

### 3.1. Patients Population

A total of 80 eyes of 40 patients affected by PsA sine-PsO were evaluated. PsA patients (72.5% women, 29/40) had a mean age 52 ± 14 years and a mean disease duration of 53.5 ± 65 months. We also recruited a HC group, consisting of 35 participants (70 eyes) matching for age (48.7 ± 13.8 years) and sex (60% women).

The mean disease activity, expressed as DAS (2.3 ± 0.8) and DAPSA (23 ± 13.6), show a disease activity status ranging between moderate and severe disease. The BASDAI score was 2.8 ± 3, VASp was 6.1 ± 1.7 and VASgh was 6.4 ± 1.6, while HAQ-SpA was 1.1 ± 0.5. At the time of the study, seven PsA patients (17.5%) showed enthesitis, eight patients (20%) presented dactylitis, while no patients had axial involvement. Laboratory indices from PsA patients revealed ESR values 13.1 ± 12 mm/h and CRP 0.4 ± 0.8 mg/dl. A cumulative dosage of PDN of 1 ± 2 g was recorded from the PsA group.

### 3.2. Ophthalmological Examinations in PsA Patients

**Figure 1:**
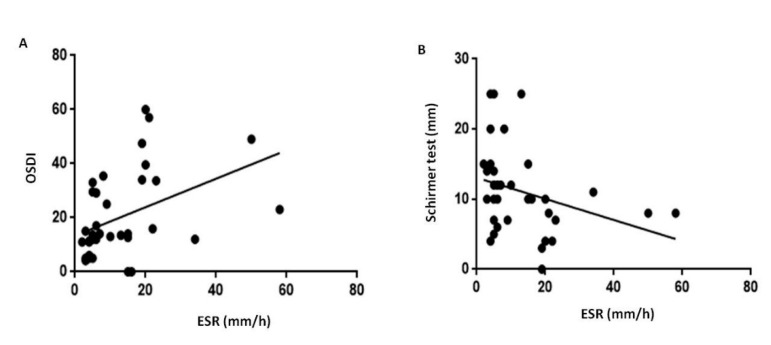
Impairment in quality and quantity of the tear film in patients with psoriatic arthritis sine psoriasis. Abbreviation: OSDI, Ocular Surface Disease Index; ESR, Erythrocyte sedimentation rate. In psoriatic arthritis patients, ESR resulted positively related with OSDI questionnaire (panel (**A**), r = 0.6, *p* < 0.001) and negatively related with Schirmer test (panel (**B**), r = −0.43, *p* = 0.007). The significance of any correlation was determined by Pearson correlation test or Spearman’s rank correlation coefficient where appropriate. *P* values < 0.05 were considered significant.

The BCVA of PsA patients were similar to those in HC group (Table 1). An abnormal OSDI was found in 60% of PsA population (Table 1). A total of 30 (75%) out of 40 PsA subjects were definitively diagnosed with dry eye (39.9% with BUT <10 sec; 23.4% with Schirmer ≤5 mm; 36.7% with both BUT <10 sec and Schirmer ≤5 mm). A positive correlation resulted between OSDI and ESR (*p* < 0.001, r = 0.6, Figure 1A). There was a significant difference in the prevalence of dry eye between controls and patients (χ^2^ = *p* < 0.0001). Interestingly, when considering Schirmer test values, a negative correlation resulted between the scale of wetness and ESR (*p* = 0.007, r = −0.43, Figure 1B).

### 3.3. Standard Automated Perimetry

**Figure 2:**
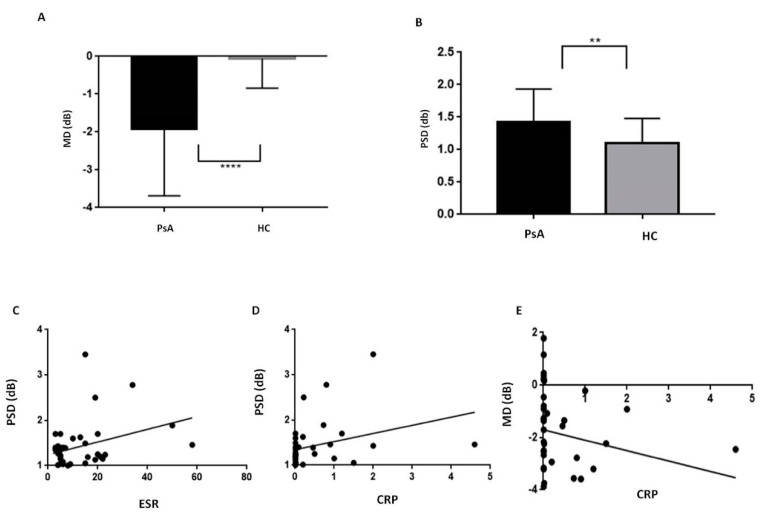
Standard Automated Perimetry in patients with psoriatic arthritis sine psoriasis. PsA, Psoriatic Arthritis; HC, healthy controls; MD, Mean Deviation, PSD, Pattern Standard Deviation; ESR, Erythrocyte sedimentation rate; CRP, C-Reactive Protein. Standard automated perimetry demonstrated in PsA patients a lower MD (panel (**A**), *p* < 0.0001) and a higher PSD (panel (**B**), *p* = 0.005) in comparison with the HC. In PsA patients, PSD was positively related with ESR (panel (**C**), r = 0.3, *p* = 0.04) and CRP (panel (**D**), r = 0.4, *p* = 0.01) while MD was related with CRP (panel (**E**), r = −0.4, *p* = 0.01). Continuous variables were compared using the parametric unpaired t-test or the nonparametric Mann–Whitney U test when appropriate. The significance of any correlation was determined by Pearson correlation test or Spearman’s rank correlation coefficient where appropriate. *P* values < 0.05 were considered significant.

Standard automated perimetry (SAP) demonstrated a higher MD and PSD in PsA patients compared with HC (*p* < 0.0001 and *p* = 0.005, respectively, Table 1, Figure 2A,B). ESR and CRP resulted in a positive correlation with PSD (*p* = 0.04 with r = 0.3, *p* = 0.01 with r = 0.4, respectively, Figure 2C,D). CRP was also correlated with MD (*p* = 0.01 r = −0.4, Figure 2E). PSD and MD did not correlate with the age, disease duration, and disease activity in PsA population. In PsA patients, visual field index (VFI) values were similar to controls (range 98–100% for both, Table 1).

### 3.4. Fundus Perimetry

**Figure 3:**
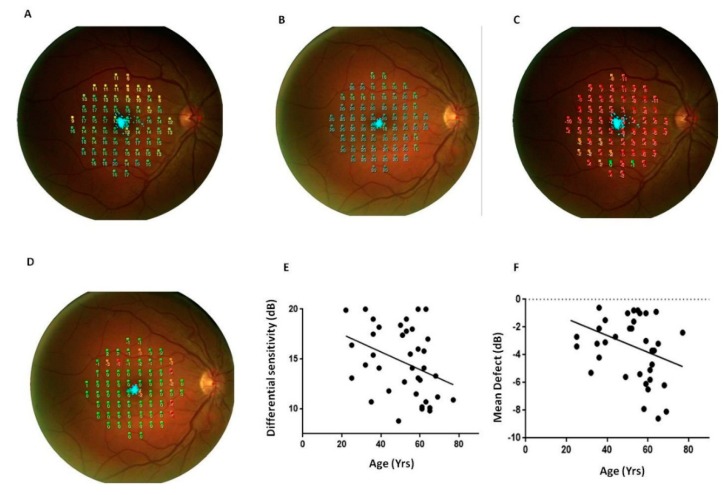
Fundus perimetry in patients with psoriatic arthritis sine psoriasis. FP, Fundus Perimetry; PsA, Psoriatic arthritis; HC, healthy control. Representative scans of the retinal differential light sensitivity and mean defect of the posterior pole were reported from a PsA patient (panels (**A**) and (**C**)) and a sex/age-matched HC (panels (**B**) and (**D**)). Panels (**E**,**F**): Differential light sensitivity and mean defect resulted related with the age (r = −0.4, *p* = 0.03, for both the correlations). The significance of any correlation was determined by Pearson correlation test or Spearman’s rank correlation coefficient where appropriate. *P* values < 0.05 were considered significant.

Figure 3 shows representative scans of the mean differential light sensitivity (Panels A–B) and mean defect (Panels C–D) measured by FP at the posterior pole from a PsA patient and a sex/age-matched HC.

**Figure 4:**
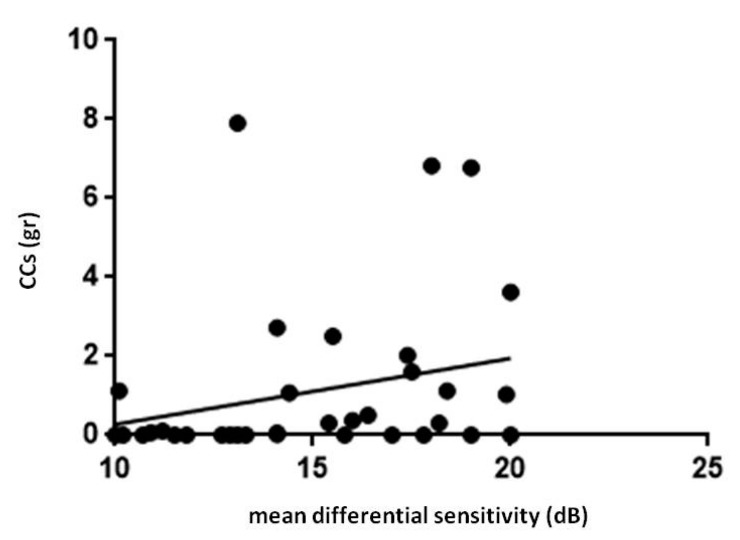
Retinal light sensitivity and glucocorticoids in patients with psoriatic arthritis sine psoriasis. FP, Fundus Perimetry; CCs, corticosteroids. In patients with psoriatic arthritis, FP mean differential sensitivity showed a positive correlation with cumulative dose of CCs (r = 0.4, *p* = 0.02). Statistical analysis was performed by Spearman correlation. *P* values < 0.05 were considered significant (* *p* < 0.05).

FP mean differential sensitivity was lower in PsA patients whereas FP mean defect values were higher in PsA patients respect to HC (*p* < 0.0001, for both the comparisons, Table 1). Differential light sensitivity resulted negatively correlated with the age while MD was directly related with age (*p* = 0.03, r = −0.4 Figure 3). A positive correlation between FP differential sensitivity and the cumulative dose of glucocorticoids occurred in PsA patients (*p* = 0.02, r = 0.4, Figure 4).

### 3.5. Spectral-Domain Optical Coherence Tomography

**Figure 5:**
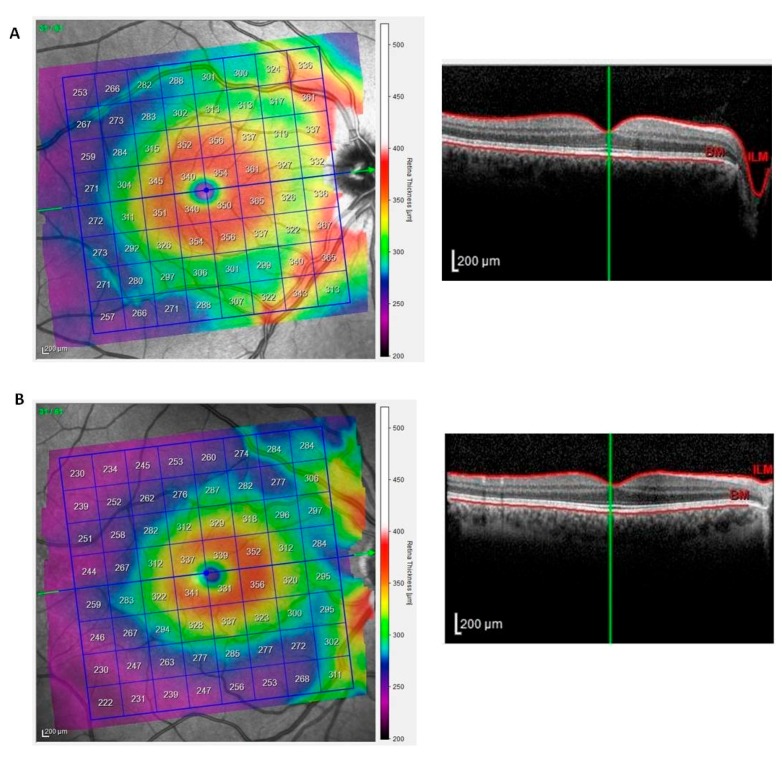
Total retinal thickness of the posterior pole in patients with psoriatic arthritis sine psoriasis. The posterior pole scanning protocol was performed with SD-OCT. Representative scans of the total retinal thickness of the posterior pole from a healthy control and a patient with psoriatic arthritis are depicted in panels (**A**,**B**). The left image shows the color-coded map of the posterior pole. The right image is the respective cross-sectional OCT scan as a grayscale image. The green line indicates the location of the cross-section.

The total retinal thickness of the posterior pole was analyzed using SD-OCT [14]. Representative scans of the total retinal thickness of the posterior pole from a HC and a PsA patient are depicted in Figure 5A,B.

The mean thickness of the posterior pole from PsA patients was similar to those from healthy eyes at the hemi-superior and hemi-inferior fields and in the total scans (Table 1).

## 4. Discussion

To the best of our knowledge, this is the first study performing a non-invasive and multimodal evaluation of ocular discomfort along with retinal function and morphology in a selected cohort of PsA patients sine-PsO who did not report visual impairment and other ocular abnormalities.

Our study found a higher prevalence of ocular discomfort, as measured by OSDI questionnaire, in patients with PsA compared to HC. Accordingly, the tear film function was significantly altered in PsA as showed by impaired BUT and Schirmer test values measured in these patients. Taken together, the tests documented a significantly elevated prevalence of dry eye in PsA patients compared with controls even in the absence of clinically evident PsO [8,9]. Moreover, a significant correlation resulted between inflammatory markers, OSDI, and the Schirmer test. These findings are consistent with the idea of multiorgan inflammatory involvement in PsA disease sine-PsO [8,31] as demonstrated in other chronic inflammatory disorders, such as PsO [8,9,10,11,12,13], diabetes mellitus [32,33], and thyreopathies [34]. In this context, the presence of dry eye might reflect the inflammatory status being an indicator of the systemic involvement in PsA [35]. The management of dysfunctional tear syndrome is still a challenge in PsA patients since without an adequate diagnosis it can result in a strong degradation in vision and thus in quality life [33]. To date, various recommended treatment options are available for several categories of dry eyes including artificial tears, gels, as well as topical anti-inflammatory and immunomodulatory agents [33]. However, future studies are needed in order to demonstrate if systemic therapies used for PsA management, such as biological therapies, can be effective also in adjusting dry eye syndrome.

Assuming that relevant signs of systemic disease may be revealed as abnormalities occurring in the eye, a clinical evaluation of retinal morphology and function should be performed in order to detect subclinical damage of the visual system early [9,32,33,34]. In addition, careful ophthalmological examination of patients with PsA sine-PsO may produce valuable clinical information on disease activity status.

On this basis, we aimed to perform visual field examinations and fundus perimetry in our cohort of PsA patients: Significant alterations of visual field indexes, such as MD and PSD, occurred in PsA patients compared with controls. By using SAP and FP, it was possible to detect functional retinal changes that can be related to systemic inflammatory response in PsA with a moderate to severe disease activity. According to the evidence, increased plasma levels of circulation inflammatory cytokines may affect retinal function and decreasing retinal sensitivity [8,9]. This study reports a significant correlation between inflammatory markers (ESR and CRP) and visual field parameters may support the hypothesis of a link between systemic inflammation and retinal changes [36,37,38,39]. Using fundus perimetry, we firstly reported subclinical abnormalities in visual function in PsA patients in terms of mean differential light sensitivity. It is noteworthy that FP and SAP measured differential light sensitivity alterations of our patients in different conditions of retinal adaptation (mesopic and photopic, respectively). This may add value to the individuation of early functional involvement of visual system in PsA sine-PsO patients.

Abnormalities in FP retinal sensitivity were found to be inversely related to age suggesting that older patients have lower retinal sensitivity function while FP Mean defect increased with increasing age. Since no correlation between age and the retinal sensitivity was observed in controls, it is likely that, in PsA patients, the abnormal retinal sensitivity function can be related to a potential damage in retinal structures detectable in chronic diseases [37,40]. A positive correlation was registered between differential light sensitivity and the cumulative dose of corticosteroids in our study population, supporting a potential protective role of steroids in maintaining retinal function in course of a systemic inflammatory disease [41]. However, it should be noted that perimetric indices of visual field can be affected by the significant ocular discomfort of PsA patients, in accordance with the literature reporting that the better tear film stability the higher optical quality of the anterior corneal surface [41]. Published studies showed that dry corneal surfaces can certainly affect the SAP findings [42,43,44]. However, SAP of PsA patients and healthy controls performed in our study can be considered equally reliable in terms of fixation errors, false positives, and negatives (Table 1). In our clinical practice, an adequate amount of tear substitute in gel (gel tear substitute or gel artificial tear) is administered in the inferior conjunctival sac of each eye from patients suffering from dry eye. Thus, when the return of a clear vision is obtained, the visual field and/or FP examinations were performed. In this way, the procedure could warrant a more regular ocular surface along with increased comfort for patients, and improvement in visual function during visual field and/or FP testing [43,44,45]. In fact, in our cohort, there were no statistically significant differences between the two groups during SAP examination in terms of fixation errors values (*p* = 0.966); false positive (*p* = 0.286), and false negative (*p* = 0.654) results.

Perimetric indices were worse in PsA than in controls even in the absence of statistically significant alterations of total retinal thickness measured at the posterior pole [14]. No studies to date have documented retinal assessment in PsA patients using SD-OCT, FP, and SAP. We firstly documented that retinal thickness measured at the posterior pole did not significantly differ between our cohort of PsA patients and healthy eyes, despite a slight decrease of the total retina thickness in the eyes of the first group. In addition, the possibility of correlating the anatomical with functional findings using SD-OCT along with FP allows ophthalmologists to measure the level of loss of retinal sensitivity associated with different structural damage and to know which retinal layer is predominantly involved in the genesis of the damage to visual function [18]. However, the lack of differences between PsA patients and HC in overall retinal thickness does not exclude early subtle retinal changes in patients. Further research of microvascular networks at the retinal level could provide more detail on subclinical structural/vascular abnormalities related to systemic inflammation. Specifically, OCT angiography (OCT-A) should be added in the multimodal eye assessment of PsA patients [38].

The results of this study suggest that the assessment of disease activity in PsA patients may be “apparently” adequate when evaluated only at joints level: Ophthalmological evaluations and the study of visual function may reveal consequences of a chronic inflammatory status which could lead to early visual loss. In particular, using either SAP or FP and OCT assessment could allow the detection of early changes in visual function even before clinically detectable retinopathy. These ancillary tests may serve as a useful monitoring tool over the entire course of the disease [36].

Evidence from our study supports the hypothesis of PsA as a systemic inflammatory disease with both autoimmune and metabolic footprints [33,46,47]. Future studies are needed to allow analysis of different retinal structures at both neural and vascular levels to give complementary information and improve our findings. A tailored ophthalmological follow-up of PsA patients can help in providing early detection of potential anatomical changes to the retina.

## Figures and Tables

**Table 1:** Ophthalmological findings from the study population.

Multimodal Assessment		PsA (*n* = 40)	HC (*n* = 35)
BCVA (logMAR)		0.01 ± 0.1	0.01 ± 0.03
**Dry Eye**	OSDI score ≥ 13	24/60	0/0
	Schirmer test ≤ 5 mm	18/45	0/0
	BUT < 10 sec	23/57.5 ***	3/8.6
	Dry eye	30/75 ****	3/8.6
**Standard** **perimetry**	Fixation errors (median; min–max)	5.00; 0.00–21.42	5.88; 0.00–18.75
	False positive (median; min–max)	0.50; 0.00–7.00	1.00; 0.00–7.00
	False negative (median; min–max)	0.00; 0.00–8.00	0.00; 0.00–8.00
	Mean deviation (dB)	−1.92 ± 1.75 ****	−0.06 ± 0.77
	PSD (dB)	1.42 ± 0.5 **	1.1 ± 0.37
	VFI (range %)	98–100	98–100
**Fundus** **perimetry**	Differential light sensitivity (dB)	14.7 ± 3.4 ****	17.44 ± 1.38
	Mean defect (dB)	−3.3 ± 2.43 ****	−1.25 ± 0.94
**SD OCT**	**Posterior Pole (µm)** -superior hemifields -inferior hemifields -total scan	295.7 ± 16.18 294.4 ± 13.6 292.9 ± 13.24	297.3± 6.37 297.6 ± 6.41 297.3 ± 7.03

Abbreviation: HC, healthy controls; PsA, Psoriatic Arthritis Patients; NA, not applicable; BCVA, best corrected visual acuity; PSD, Pattern Standard Deviation; VFI, visual field index; SD-OCT, Spectral Domain Optical Coherent Tomography. Continuous variables were shown using mean and standard deviation (SD) while categorical variables with absolute frequencies and percentages. Values from patients were compared with controls using the parametric unpaired t-test or the nonparametric Mann–Whitney U test when appropriate and *p* values < 0.05 were considered significant (** *p* < 0.01, *** *p* < 0.001, **** *p* < 0.0001 compared to controls).
